# Horticultural Characteristics of Summer Apple Cultivars from Turkey

**DOI:** 10.3390/plants11060771

**Published:** 2022-03-14

**Authors:** Kerem Mertoğlu, Emre Akkurt, Yasemin Evrenosoğlu, Ayşen Melda Çolak, Tuba Esatbeyoglu

**Affiliations:** 1 Department of Horticulture, Faculty of Agriculture, Eskisehir Osmangazi University, 26160 Eskisehir, Turkey; emre.akkurt13@gmail.com (E.A.); yevrenosoglu@ogu.edu.tr (Y.E.); 2 Department of Food Development and Food Quality, Institute of Food Science and Human Nutrition, Gottfried Wilhelm Leibniz University Hannover, Am Kleinen Felde 30, 30167 Hannover, Germany; 3 Department of Horticulture, Agricultural and Natural Science Faculty, Usak University, 64000 Usak, Turkey; aysenmelda.colak@usak.edu.tr

**Keywords:** antioxidant, climate change, *Malus domestica*, polyphenol, vitamin C

## Abstract

Early varieties with a short active vegetation period are not exposed to abiotic stress factors for instance drought, high temperature, etc., caused by global climate change, and biotic stress factors such as codling moth (*Cydia pomonella*) and apple scab (*Venturia inequalis*) that cause serious economic losses. Therefore, their places are extremely important regarding sustainable, economical and quality production. In this context, four early varieties of apple (‘Vista Bella’, ‘Summer Red’, ‘Williams Pride’ and ‘Jersey Mac’) grown in Eskisehir—Turkey where has continental climate were characterized in terms of horticultural characteristics. The difference observed in flowering and harvest times between years showed that average air temperatures are essential on phenological characteristics. Furthermore, high temperature accelerates development physiology of fruit. Major important characteristics, namely, fruit length, width, weight, soluble solid content, antioxidant activity, vitamin C and total phenolic content were found in the range of 39.19–50.88 mm, 51.48–60.81 mm, 80.59–86.16 g, 11.87–12.19%, 50.69–80.57%, 2.24–4.79 mg 100 mL^−1^ and 259.23–871.42 mg GAE (gallic acid equivalent) L^−1^, respectively. ‘Summer Red’ and ‘Williams Pride’ varieties were found to be good in terms of pomological characteristics, while ‘Vista Bella’ came to the fore with its chemical properties. On the other hand, ‘Jersey Mac’ has shown superior performance in terms of both pomological and chemical characteristics. Pomological characteristics are affected more by ecological differences, while chemical characteristics vary according to the changes in the pomological properties. Pomological characteristics were found to have a significant positive correlation with each other but negatively correlated with chemical characteristics.

## 1. Introduction

On a global scale, apple is by far the first among the deciduous fruit species in terms of production area (approximately 5 Mio decare) and production quantity (approximately 90 Mio tons). While about 9 Mio tons of apples are traded between countries as fresh, over 3 Mio liters of fruit juice is exported between countries. In addition to being favorably consumed as fresh and dry, apple is also used in different branches of the industry as processed and these give a special place to apple in the world market [1].

However, changes in ecological factors due to global climate change make apple cultivation less sustainable and economical [2,3]. It is reported that the burns caused by light and temperature in late varieties, cultivation of which is left to the period when high temperatures prevail, lead to serious marketing and storage problems [4,5]. It was also reported that the struggle against the *Cydia pomonella* and *Venturia inequalis*, epidemics of which peaked during this period was ineffective and residue problems were experienced due to intense (about 20 times in the season) chemical management [6]. These situations increase the production cost while reducing the market value [7].

As a matter of fact, if horticulture facilities are established with early varieties and the harvest is completed before the risky period, the possible effects of these problems will be mitigated. Early varieties with a short active vegetation period also allow farmers to devote time to different jobs. In addition, it is reported that the acidity is high in early varieties and this condition triggers positive effects for human health [8]. It is stated that by processing these products with high acidity in industry, the activity of microorganisms in the products is limited and the stability of the product is maintained [9]. Early varieties can be marketed at reasonable prices in the domestic market during the beginning of the production season, when the crop diversity is low, and it is, also, crucial that they increase the country’s export potential [10]. Based on all these reasons, it is emphasized that the early varieties should be developed, and their cultivation should be extended [11,12,13,14].

*Cydia pomonella* preffered to oviposit late ripening apple cultivars than in the early maturing ones [15]. Sunburn damage on apple surface and flesh was higher in cultivars ripening in the high temperature period [16]. Early apple varieties have a low values in terms of fruit mass characteristics due to the short period from flowering to harvest [17]. ‘Jersey Mac’ was the only early variety and found as a lightest one with 112.3 g among studied varieties and it is reached to harvest maturity in 89 days after full bloom. On the other hand, early cultivars contain higher biochemical substances per unit area thanks to their lower intercellular spaces. ‘Jersey Mac’ was the top cultivar in terms total phenolic content (494.8 mg GAE L^−1^), vitamin C (5.63 mg 100 mL^−1^) and antioxidant activity (78.23%) [8].

This study, which was carried out in 2018 and 2019, was conducted to assess the phenological and physico-chemical properties of certain summer apple varieties in the ecological conditions of Eskişehir (Turkey). In addition, it was indicated how the growing season affects the physiology of fruits and trees and the interactions between the studied characteristics were also revealed.

## 2. Results

### 2.1. Phenological Observations

Phenological characteristics belonging to each variety are given in Table 1. Accordingly, the full blooming times of the ‘Williams Pride’, ‘Summer Red’ and ‘Vista Bella’ were recorded on 9 April in 2018 and, respectively, on 23, 24 and 27 April in 2019. The full blooming of ‘Jersey Mac’ was observed on 12 April in 2018 and on 27 April in 2019. Harvests of varieties were made approximately 20 days earlier due to warmed earlier and remained higher air temperatures in 2018. In addition, all varieties reached to harvest maturity in a shorter time after full blooming in 2018 (Table 1).

### 2.2. Physico-Chemical Parameters

All characteristics differed statistically due to year or variety except stalk width. The effect of cultivar was found to be more critical on the variation in fruit width, fruit length, stalk length, seed number, lightness (L*) and peel hue (h°), while year contributed more to the total variation in fruit weight and and chroma (C*) (F values were given in brackets) (Table 2). In addition, interaction effect of factors on some characteristics was found to be important (Table 2).

‘Jersey Mac’ (60.81 cm) and ‘Williams Pride’ (60.06 cm) varieties’ have widest fruit and statistically constitute the upper group, while the fruits of ‘Vista Bella’ (51.48 cm) take into the lowest group. ‘Summer Red’, ‘Williams Pride’ and ‘Jersey Mac’ varieties have 50.88 cm, 49.60 cm and 47.96 cm values in terms of fruit length, respectively, and did not differ from each other. ‘Vista Bella’ followed these varieties with 39.19 cm. While unimportant differences were obtained between the years with regard to fruit width, the average fruit length and fruit weight were found to be higher in 2018 (48.24 cm and 89.87 g, respectively) than in 2019 (45.57 cm and 77.51 g, respectively). Stalk length of the varieties differed between 12.50 mm (Vista Bella) and 20.28 mm (Williams Pride) and the average stalk length of 2018 (19.19 mm) was found to be higher than the average of 2019 (17.11 mm).

While there was no difference in the number of seeds per fruit between years difference between the varieties was found to be significant where the ranking was as follows: ‘Jersey Mac’ (7.41 seeds/fruit) > ‘Williams Pride’ (6.10 seeds/fruit) > ‘Summer Red’ (5.04 seeds/fruit) > ‘Vista Bella’ (4.64 seeds/fruit).

Lightness (L*) and chroma (C*) values of over peel color were statistically significant among varieties and years. Average values of both these characteristics were higher in 2018 (L*—48.95; C*—35.32) compared to 2019 (L*—46.32; C*—32.12). ‘Summer Red’ had the highest L* (51.95), while C* was found to be the highest in the ‘Vista Bella’ (36.43). Peel hue (h°) of the varieties did not differ significantly between years. In comparison, the difference between the varieties was found to be statistically significant. Whereas the highest h° value was measured from ‘Summer Red’ (37.03), the lowest was found in the ‘Vista Bella’ variety (24.07) (Table 2).

Chemical properties of the four varieties are shown in Table 3. While there was no statistical significance between the varieties in terms of soluble solid content (SSC), the effects of cultivar, year and cultivar*year interaction were found to be significant in all other characteristics examined.

Whereas the difference between years in terms of SSC value was statistically significant; no significant difference was found among varieties. In 2018, SSC (12.29%) was found to be higher than 2019 (11.72%). As expected, pH value was detected as low (3.22) in 2018 when TA was high (1.23%) and pH value was high (3.26) in 2019 when TA was low (0.90%). Similarly, pH and TA were showed significant differences between varieties and ‘Jersey Mac’ with the highest TA (1.17%) had the lowest pH (3.16). Conversely, ‘Williams Pride’ with the lowest TA (0.88%) had the highest pH (3.40). When the vitamin C and TPC of the varieties were examined, it was seen that the highest amounts with regard to both characteristics were measured in ‘Jersey Mac’ as 4.79 mg 100 mL^−1^ and 871.42 mg GAE L^−1^, respectively. These compounds, which show high antioxidant properties, have enabled to stand out ‘Jersey Mac’ variety in terms of antioxidant activity (80.57%). While lowest vitamin C was obtained from ‘Summer Red’ with 2.24 mg 100 mL^−1^. Similar results were measured in ‘Williams Pride’ in terms of TPC and antioxidant activity with 259.23 mg GAE L^−1^ and 50.69%, respectively. On the other hand, vitamin C, which was measured as 2.66 mg 100 mL^−1^ in 2018, increased to 3.77 mg 100 mL^−1^ in 2019. Oppositely, TPC and antioxidant activity properties, which were detected as 594.39 mg GAE L^−1^ and 79.30% in 2018, decreased to 431.67 mg GAE L^−1^ and 56.02%, respectively.

### 2.3. Correlation among Properties and PCA

Relations among characteristics are given in Table 4 with their correlation coefficients. A high degree of positive relation was found between fruit length and fruit width (0.77 ***). In addition, fruit weight characteristics showed a positive increase depending on these two characteristics, and the correlation coefficients were determined to be 0.68 *** and 0.71 ***, respectively. In the study, SSC was found to be in a negative relation with fruit weight, width and length characteristics respectively at −0.22 **, −0.20 ** and −0.16 *. Similarly, significant negative relations were found between fruit length with vitamin C (−0.19 *) and TPC (0.19 *), fruit weight with titratable acidity (−0.16 *) and antioxidant activity (−0.14 *). An increase in the number of seeds per fruit allowed an increase in the width (0.3 *), length (0.32 *) and weight (r: 0.16 *) of the fruit. While stalk width was found to be ineffective on physical properties, stalk length had a significant effect on the increase in fruit width (0.40 ***), length (0.46 ***) and weight (0.13 *).

PCA was conducted to obtain a broad view on the correlations between physico-chemical characteristics and determination of the variation sources. Same way was used in lots of *Rosaceae* members such as cherries [18], quince [19] and apricot [20], including apple [21]. According to the obtained results, observed variability was explained by seven components. The first three components explain 77.6% of the total observed variability (Table 5). PC1 represents fruit width (FW), stalk length (SL) and fruit length (FL) and explains 35.30% of the total variation in the data set while PC2 explained 26.2% with titratable acidity (TA) and total phenolic content (TPC). This can be interpreted as the fact that variation caused by cultivar and year occurs largely in physical properties at first, whereas chemical properties come later. PCA results also mean that the most sensitive characteristics to ecological differences are identified traits that were found in PC1 (FW, SL and FL) and PC2 (TA, TP).

**Figure 1:**
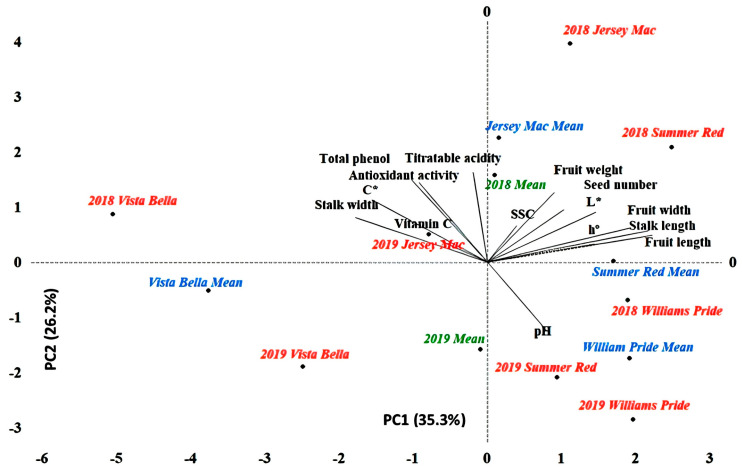
Bi-plot segregation of physico-chemical characteristics along with cultivars.

PCA is an effective way to identify the cultivars with superior quality performance according to investigated characteristics. In this context, when the averages of the two-year data were examined, higher values belonging to pomological characteristics were obtained from ‘Summer Red’ and ‘Williams Pride’ varieties, while chemical properties were found at higher level in ‘Vista Bella’. On the other hand, ‘Jersey Mac’ has shown superior performance in terms of both physical and chemical properties (Figure 1). However, ‘Jersey Mac’ was shown to be highly sensitive to changing ecological factors, giving results in different aspects of the PC1 plane over the years. While no such condition is observed in other varieties, it has been found that they exhibit a stable course.

## 3. Discussion

Early bloom in 2018 is thought to have been caused by April’s high temperature average (Table 6). As a matter of fact, it was stated by Szabo et al. [22] and Legave et al. [23] that supplying the temperature requirement is the most important factor affecting the onset of blooming and the linear regression equation between temperature and blooming was reported as R² = 0.55. Furthermore, it is reported that the increase in air temperature accelerates the physiology of fruit development, while this period increases in cooler weather conditions [24]. Shorter period from full bloom to harvest in 2018, than 2019 is thought to be mainly due to this physiological reaction.

Optimal weather during blooming and fruit setting periods allows the production of higher amounts of gibberellic acid and cytokinin in growth cones [25]. Thus, the number of cells in the growth cones increases and this enable larger fruit stalk, sizes and weight. The fact that in the first year of the study, the period of blooming and fruit setting coincided with the period when weather conditions were more appropriate (Table 6), is thought to have caused better results. A similar situation has been reported by Bozbuğa and Pırlak [26].

As expected, in the apple varieties with gametophytic incompatibility due to their S-alleles [27], seed number remains stable especially under controlled hybridizations [28]. Seed is one of the production centers of hormones that promote growth and development and is one of the important factors that directly affect the quality and quantity of fruit [29]. In this context, it is stated that as a result of successful pollination and fertilization, a minimum of five seeds should be found in the apple fruit for superior quality [30]. In this context, all our combinations seem suitable for each other.

Skin color is the preferred one which determines a cultivar’s market acceptance [31]. Over peel color findings of the study are compatible with Felicetti and Schrader [32] who reported considerable over peel color differences among many apple cultivars due to their genomic differences. On the other hand, it is well known that suitable temperature accelerates the accumulation of anthocyanins by the regulating expression genes of the anthocyanin biosynthetic pathway and leads to peel color differences by years [33]. In plants, after fertilization, an increase in the number of cells is observed, followed by cell expansion. During the cell expansion phase, the combination of horizontal and longitudinal development in the cell occurs simultaneously. The increase in the volume of the cells that make up the fruit, increases the weight. In these respects, a strong positive relations were determined between fruit pomological characteristics. Similar findings have also been reported in studies conducted with different varieties of apples [34,35]. As the increase in volume and weight of the fruit, increases the intercellular space. So, the accumulation occurring in the unit area decreases [32]. For this reason, it is reported that the pomological and chemical properties of the fruit are generally in a negative relation with each other [36,37].

Seed is one of the organs where auxin, cytokinine and gibberellic acid are produced in abundance, which positively affects fruit size by increasing cell number and expansion. It is also noted that these hormones also increase the transport of plant nutrients into fruit [31]. Study results were in parallel. These findings are in agreement with Buccheri and Di Vaio [38] who reported an increase in fruit sizes and weight in parallel to seed number increase.

The change in pedicel just before and after the fruit set when the hormonal signaling and production are highly seen is very important in the nutrition of the fruit in the remaining time [39]. In this process, the elongation of the pedicel axis increases the transport of assimilates produced in the plant to the fruit. As a matter of fact, positive correlations between the stalk sizes and fruit were reported in various fruit species, including the apple such as the present study [40,41].

An increase in the pH value occurs due to the breakdown of organic acids that carry the H^+^-ion. In the study, detection of a high degree of negative correlation between pH and TA is parallel to this situation (−0.50 ***). The negative relation between pH and TA in apples is reported by Mertoglu and Evrenosoglu [8] as −0.81 *** and by Eskimez et al. [34] as −0.78 ***. Ascorbic acid, an organic acid and the active ingredient of vitamin C, and phenolic compounds that make up total phenol have an acidic character, and it is noted that as the acidity of the environment increases, the amount of these compounds increases as well [8]. However, in this study, statistical significance was determined only between TPC and titratable acidity (0.49 ***). The pH value, which has negative correlation with TA, was found to negatively correlate with both vitamin C (−0.24 **) and total phenolic content (−0.48 ***). Similar findings were noted in diverse fruit species, including apples [8,35,42]. Although, both organic and phenolic acids have a high antioxidant effect [43], a statistically significant difference was only observed between antioxidant activity and the total phenolic content (0.60 ***) in the study. It is believed that this is caused by the fact that phenolic compounds mainly contribute to the antioxidant activity, and the amount of ascorbic acid is much lower than phenolic compounds. The findings are in parallel with the studies conducted by Polat et al. [37] and Mertoglu et al. [44].

The increase of coloring pigments in fruits makes the fruit darker and dull at the same time. For this reason, it was found that C* is negatively related with L* and h° values and the correlation coefficients between them were found to be −0.20 ** and −0.41 ***, respectively. In addition, it was found that the C* value has an increasing effect on fruit weight (0.24 **). This relation may have been caused by the fact that coloring agents are synthesized during photosynthesis, which also improves the physical properties of the fruit [34].

## 4. Materials and Methods

### 4.1. Apple Cultivars

In the study, four early varieties namely; ‘Vista Bella’, ‘Jersey Mac’, ‘Summer Red’ and ‘Williams Pride’ cultivated commonly all over the world which were located on the experimental field of Eskişehir Osmangazi University, Faculty of Agriculture (Turkey) and planted in 2008 with 4 m × 3 m planting ranges as grafted to ‘MM106′ rootstock were used as study material. The samplings were made from healthy and homogenous trees and annual cultural practices were performed routinely.

In Eskişehir where the study material is located, a typical continental climate prevails, and the climatic characteristics observed at the trial site during the study period are given in Table 6. It was observed that in both trial years, temperatures were higher than the long-term average due to global climate change. The increase in air temperatures also increases the potential for water vapor dissolution in the air, and therefore, a similar trend has been observed in air humidity. When the long years data were examined in terms of rainfall, there was consistency between the months of the seasons, while irregular rainfall and dry periods were observed in the years covering the study.

### 4.2. Phenological Observations

The period when flower buds bloom at a rate of 70–80% was recorded as the full blooming time. Harvest of the cultivars was made through taste, color and the occurrence of abscission layer of fruits. The total number of days between the full blooming date and the harvest date was calculated and thus required time for maturity was revealed as a day [45].

### 4.3. Pomological Characteristics

Harvested fruits were rapidly brought to the laboratory. Fruit weight was defined by an electronic scale (CPA 16001S; Sartorius, Göttingen, Germany) and to determine fruit width, fruit length, stalk width and stalk length, digital calipers were used. A colorimeter (NR20XE, Shenzhen 3nh Technology Co. Ltd., Shenzhen, China) was used to detect the color of the over peel of the fruits [46]. Seeds were taken out and counted for each fruit.

### 4.4. Determination of Chemical Characteristics

Fruits were squeezed with a juice extractor to obtain their juices. These juices were used for the phytochemical analyses. Phytochemical analyses were conducted when the last cultivar was harvested each year. The juices of the previously harvested cultivars were stored at −20 °C.

Soluble solid content (SSC) was determined with a digital refractometer (Atago PR-32α, Tokyo, Japan), and the results were expressed as percentage [45]. Titratable acidity and vitamin C contents of juices were determined by titration methods according to Spinola et al. [47], respectively.

Total polyphenol content was determined using the colorimetric Folin–Ciocalteu assay [48]. Gallic acid was used as an external standard for the calibration curve and the results were expressed in terms of mg of gallic acid equivalents per liter (mg GAE L^−1^) of fruit juice.

DPPH method was chosen for the determination of antioxidant activities. Primarily, 50% inhibition sample concentration (IC_50_) was calculated by drawing percent inhibition against the sample concentrations. Then, up to IC_50_ value samples were taken and the ability to remove the DPPH radical was determined according to the method specified by Mertoglu et al. [44].

### 4.5. Statistical Analysis

This experiment was performed as factorial experiment by taking cultivars and years as a factor in a completely randomized design with five repetitions. In pomological features, measurements were made on twenty fruits collected from four sides of each tree (5 trees for each variety, 100 fruits). For phytochemical analyses, the same sampling method was conducted. Data were subjected to analysis of variance (ANOVA) using the GLM procedure in the statistical analysis software (Minitab-17). Significant differences among treatments were determined by using the Tukey multiple comparison test at *p* < 0.05. Relations among the investigated characteristics were revealed as a result of correlation analysis and expressed with Pearson correlation coefficients. Further, variation sources, all factors and characteristics relations were determined by principal component analysis (PCA) [49].

## 5. Conclusions

In the scope of this study, four early apple varieties were characterized in terms of phenological and physico-chemical properties. Varieties need 80 (Vista Bella) and 97 (Summer Red) days from bloom to harvest for maturity. Summer Red and Williams Pride varieties were showed good pomological properties, while Vista Bella was good in terms of chemical characteristics. Jersey Mac was placed in middle with regards to these characteristics. Seed count and C* value of peel have been found to interact with many features and have a significant impact on quality.

## Figures and Tables

**Table 1:** Phenological characteristics’ times of cultivars according to years.

	Full Blooming		Harvest		DAFBH	
	2018	2019	2018	2019	2018	2019
Williams Pride	09/04	23/04	08/07	26/07	91	94
Summer Red	09/04	24/04	13/07	30/07	96	97
Vista Bella	09/04	27/04	27/06	17/07	80	84
Jersey Mac	12/04	27/04	02/07	21/07	82	85

DAFBH: day after full bloom to harvest.

**Table 2:** Distribution of pomological characteristics by cultivars and years.

	Fruit Width (mm)	Fruit Length (mm)	Fruit Weight (g)	Stalk Width (mm)	Stalk Length (mm)	Seed Number	L*	C*	h°
Jersey Mac	60.81 ^A^	47.96 ^A^	85.55 ^ns^	3.28 ^ns^	19.74 ^A^	7.41 ^A^	48.30 ^B^	33.84 ^B^	32.04 ^AB^
Williams Pride	60.06 ^A^	49.60 ^A^	86.16	2.82	20.28 ^A^	6.10 ^B^	46.46 ^BC^	31.34 ^C^	27.98 ^BC^
Summer Red	57.00 ^B^	50.88 ^A^	82.47	2.78	19.89 ^A^	5.04 ^BC^	51.95 ^A^	33.27 ^BC^	37.03 ^A^
Vista Bella	51.48 ^C^	39.19 ^B^	80.59	3.44	12.50 ^B^	4.64 ^C^	43.84 ^C^	36.43 ^A^	24.07 ^C^
2018	59.94 ^ns^	48.24 ^A^	89.87 ^A^	3.19^ns^	19.09 ^A^	5.89 ^ns^	48.95 ^A^	35.32 ^A^	31.12 ^ns^
2019	56.74	45.57 ^B^	77.51 ^B^	2.97	17.11 ^B^	5.70	46.32 ^B^	32.12 ^B^	29.44
Cultivar (C)	*** (29.79)	*** (43.38)	ns	ns	*** (44.91)	*** (16.00)	*** (11.82)	*** (10.58)	*** (8.26)
Year (Y)	ns	** (11.32)	*** (16.28)	ns	*** (12.62)	ns	** (7.47)	*** (23.48)	ns
C*Y	*** (8.80)	*** (7.97)	* (3.43)	Ns	*** (8.70)	* (2.89)	ns	ns	ns

Means in columns with the different letters differ from each other according to Tukey’s test at * *p* < 0.05, ** *p* < 0.01, and *** *p* < 0.001, respectively. ns: non-significant.

**Table 3:** Distribution of chemical characteristics by cultivars and years.

	SSC (%)	TA (%)	pH	Vitamin C (mg 100 mL^−1^)	TPC (mg GAE L^−1^)	AA (%)
Jersey Mac	11.87 ^ns^	1.17 ^A^	3.16 ^C^	4.79 ^A^	871.42 ^A^	80.57 ^A^
Williams Pride	12.19	0.88 ^C^	3.40 ^A^	2.59 ^C^	259.23 ^C^	50.69 ^D^
Summer Red	12.02	1.16 ^A^	3.18 ^BC^	2.24 ^D^	301.98 ^C^	61.64 ^C^
Vista Bella	11.94	1.05 ^B^	3.21 ^B^	3.25 ^B^	619.50 ^B^	77.74 ^B^
2018	12.29 ^A^	1.23 ^A^	3.22 ^B^	2.66 ^B^	594.39 ^A^	79.30 ^A^
2019	11.72 ^B^	0.90 ^B^	3.26 ^A^	3.77 ^A^	431.67 ^B^	56.02 ^B^
Cultivar (C)	ns	*** (65.93)	*** (74.36)	*** (191.16)	*** (539.33)	*** (353.79)
Year (Y)	** (11.41)	*** (368.02)	* (5.39)	*** (186. 65)	*** (168.52)	*** (918.60)
C*Y	*** (7.38)	*** (54.80)	*** (8.79)	*** (10.6)	* (3.44)	*** (294.54)

SSC: soluble solid content, TA: titratable acidity, TPC, total phenol content and AA: antioxidant activity. Means in columns with the different letters differ from each other according to Tukey’s test at * *p* < 0.05, ** *p* < 0.01, and *** *p* < 0.001, respectively. ns: non-significant.

**Table 4:** Correlation coefficients among the investigated characteristics.

	Fwi	FL	Few	SWi	SL	SN	L*	C*	h°	SSC	TA	pH	Vit C	TPC
FL	0.77 ***													
FWe	0.71 ***	0.68 ***												
SWi	ns	ns	ns											
SL	0.40 ***	0.46 ***	0.13 *	ns										
SN	0.31 ***	0.32 ***	0.16 *	ns	0.34 ***									
L*	ns	ns	−0.17 *	ns	0.14 *	ns								
C*	ns	ns	0.24 **	ns	ns	ns	−0.20 **							
h°	ns	ns	−0.20 **	ns	ns	ns	0.68 ***	−0.41 ***						
SSC	−0.16 *	−0.20 **	−0.22 **	ns	ns	0.56 ***	ns	0.18 *	ns					
TA	ns	ns	−0.16 *	ns	ns	0.17 *	0.12 ^*^	0.29 **	ns	0.40 ***				
pH	ns	0.14 *	ns	ns	0.12 *	0.12 *	ns	ns	ns	ns	−0.50 ***			
Vit C	ns	−0.19 *	ns	ns	−0.12 *	0.15 *	−0.16 *	ns	ns	ns	Ns	−0.24 **		
TPC	ns	−0.14 *	Ns	ns	ns	0.18 *	ns	0.18 *	ns	ns	0.49 ***	−0.48 ***	0.55 ***	
AA	ns	ns	−0.14 *	ns	ns	ns	ns	0.33 ***	ns	ns	0.37 ***	−0.28 **	ns	0.60 ***

FL: fruit length, FWe: fruit weight, FWi: fruit width, SL: stalk length, SN: seed number, SSC: soluble solid content, SWi: stalk width, TA: titratable acidity, Vit C: vitamin C, TPC: total phenolic content and AOA: antioxidant activity. *, ** and ***: correlations are significant at *p* < 0.05, *p* < 0.01 and *p* < 0.001, respectively. ns: non-significant.

**Table 5:** Eigenvalues and total variability proportion of principal component (PC) axes for investigated characteristics.

	PC1	PC2	PC3	PC4	PC5	PC6	PC7
Fruit Width	0.360	0.157	−0.283	−0.111	−0.007	−0.029	0.135
Fruit Length	0.419	0.123	0.051	−0.009	−0.038	0.043	−0.157
Fruit weight	0.169	0.320	0.058	−0.445	−0.068	−0.337	−0.300
Stalk Width	−0.337	0.206	−0.022	−0.084	0.351	0.596	0.079
Stalk Length	0.410	0.148	−0.059	−0.035	−0.086	0.059	0.217
Seed Number	0.193	0.242	−0.474	−0.091	0.049	0.199	0.011
L*	0.275	0.231	0.279	0.287	−0.090	0.342	0.030
C*	−0.294	0.289	0.197	−0.113	−0.284	0.117	−0.339
h°	0.272	0.082	0.122	0.502	0.146	0.187	−0.376
SSC	0.073	0.168	0.297	−0.410	0.550	0.024	−0.255
Titratable Acidity	−0.037	0.415	0.288	0.092	0.235	−0.183	0.612
pH	0.150	−0.315	−0.096	−0.450	−0.078	0.460	0.169
Vitamin C	−0.120	0.099	−0.532	0.208	0.325	−0.063	−0.264
Total phenolic content	−0.195	0.382	−0.298	0.036	−0.012	−0.151	0.132
Antioxidant activity	−0.174	0.369	−0.045	−0.041	−0.530	0.216	−0.045
Expl.Var	5.289	3.933	2.419	1.995	1.010	0.261	0.091
Prp.Totl	0.353	0.262	0.161	0.133	0.067	0.017	0.006
Cumulative—%	35.3	61.5	77.6	90.9	97.6	99.4	100

Red means that characteristic mostly interacts under related PC.

**Table 6:** Climatic data of research area in 2018 and 2019 years.

	Precipitation (mm)			Humidity (%)			Temperature (°C)		
	2018	2019	Long Term	2018	2019	Long Term	2018	2019	Long Term
March	53.6	9.2	30.3	73.5	64.5	65.1	9.2	6.3	5.3
April	12.6	24.8	40.5	61.6	69.3	62.8	13.8	9.5	9.9
May	62.2	39.8	41.9	74.8	65.1	60.8	16.8	16.5	14.8
June	46.6	36.6	29.9	69.5	67.9	57.2	19.9	20.9	18.9
July	39.2	36.4	14.2	65.5	62.4	53.0	22.3	21.3	21.9
August	18.0	3.2	12.4	63.5	61.0	54.7	22.9	22.3	21.8
September	2.8	4.0	17.0	65.4	62.1	58.4	18.6	18.3	17.3

## Data Availability

No new data were created or analyzed in this study. Data sharing is not applicable to this article.
